# Circulating SIRT1 and Sclerostin Correlates with Bone Status in Young Women with Different Degrees of Adiposity

**DOI:** 10.3390/nu14050983

**Published:** 2022-02-25

**Authors:** Rossella Tozzi, Davide Masi, Fiammetta Cipriani, Savina Contini, Elena Gangitano, Maria Elena Spoltore, Ilaria Barchetta, Sabrina Basciani, Mikiko Watanabe, Enke Baldini, Salvatore Ulisse, Carla Lubrano, Lucio Gnessi, Stefania Mariani

**Affiliations:** 1 Department of Molecular Medicine, “Sapienza” University of Rome, 00161 Rome, Italy; rossella.tozzi@uniroma1.it; 2 Department of Experimental Medicine, Section of Medical Physiopathology, Food Science and Endocrinology, “Sapienza” University of Rome, 00161 Rome, Italy; davide.masi@uniroma1.it (D.M.); fiammetta.cipriani@uniroma1.it (F.C.); savi.86@hotmail.it (S.C.); elena.gangitano@uniroma1.it (E.G.); mariaelena.spoltore@uniroma1.it (M.E.S.); ilaria.barchetta@uniroma1.it (I.B.); sabrina.basciani@uniroma1.it (S.B.); mikiko.watanabe@uniroma1.it (M.W.); carla.lubrano@uniroma1.it (C.L.); lucio.gnessi@uniroma1.it (L.G.); 3 Department of Surgical Sciences, “Sapienza” University of Rome, 00161 Rome, Italy; enke.baldini@uniroma1.it (E.B.); salvatore.ulisse@uniroma1.it (S.U.)

**Keywords:** obesity, adipose tissue, SIRT1, sclerostin, bone mineral density (BMD), trabecular bone score (TBS)

## Abstract

Sirtuin1 (SIRT1) and sclerostin play important roles in adipose tissue and bone metabolism. We evaluated the circulating SIRT1 and sclerostin relationship with mass and quality of bone while considering the degree of adiposity. Sixty-six premenopausal women (16 underweight, 25 normal weight and 25 with obesity), aged <50 years, were enrolled. Plasma SIRT1, sclerostin and DXA body composition (total fat mass (FM), abdominal visceral adipose tissue, lean mass, trabecular bone score (TBS) and lumbar spine and femoral neck (FN) bone mineral density (BMD)) were assessed. The patients with obesity showed the lowest SIRT1 and TBS values and the highest sclerostin concentrations; BMD increased with FM and BMI and had an inverse association with SIRT1. Sclerostin was negatively correlated with SIRT1 (ρ = −0.37, *p* = 0.002). When spine BMD, FN BMD and TBS were standardized for BMI, a positive correlation with SIRT1 and a negative correlation with sclerostin were seen (*p* < 0.005). In the regression analysis, sclerostin was the best independent, negative predictor for BMD and TBS, while SIRT1 directly predicted TBS (*p* < 0.05). In conclusion, blood measurement of SIRT1 and sclerostin could represent a snapshot of the bone status that, taking into account the degree of adiposity, may reduce the interference of confounding factors in the interpretation of bone health parameters.

## 1. Introduction

Bone mass loss, as occurs in osteopenia and osteoporosis, is one of the most common metabolic bone disorders [1]. Its prevention, which is a global health priority, focuses on population stratification and early treatment of the most predisposed subjects. However, osteoporosis remains largely underdiagnosed and undertreated because it is clinically silent until it manifests in fractures [2]. Many factors have been associated with a higher risk of bone demineralization, including age, prolonged glucocorticoids treatment or estrogen deprivation. The impact of obesity on bone mass is still controversial [3]. Weight excess has been traditionally linked to bone protection thanks to the observed increase in bone mineral density (BMD) through the mechanical load. However, obesity is associated with an increased risk of fracture in some reports, possibly due to the trabecular bone metabolic and microcirculatory derangements responsible for microarchitecture alteration [4,5,6]. In this regard, the trabecular bone score (TBS) has recently emerged as a useful clinical parameter of bone microarchitecture degradation and is found to be particularly sensitive to glycometabolic alterations [7]. The pathophysiology underlying the link between bone and fat, as well as its possible means of evaluation, are therefore still a matter of debate, and several molecules possibly involved were recently described.

Sirtuins (SIRTs) are a class of nicotinamide adenine dinucleotide (NAD)-consuming enzymes implicated in several biological pathways. Through epigenetic regulation, SIRTs are able to mediate cellular activities, both in prokaryotes and eukaryotes. Among the seven SIRTs known, SIRT1 is the most studied. It plays important roles in energy metabolism, food intake, fat mobilization and muscle differentiation [8], and most studies have revealed that as adiposity increases, SIRT1 expression decreases, both in peripheral tissues and blood [9,10,11]. Although recent studies indicate that SIRT1 may protect against the metabolic decline associated with senescence, including aging-related bone loss [8,12,13], the participation of circulating SIRT1 in skeletal homeostasis has not been fully elucidated. SIRT1 seems to be involved in the fate of mesenchymal stem cells (MSC) by promoting osteogenic differentiation and suppressing adipogenic differentiation [14,15]. Interestingly, the pharmacological induction of SIRT1 expression improves bone quality, both in murine and human models. Indeed, mice treated with the SIRT1-activating compound SRT1720 show a significant increase in bone mass [16]. In this regard, Artsi and colleagues demonstrated that a 6-week oral administration of a SIRT1 activator can reduce ovariectomy-induced bone loss and biomechanical deterioration in adult C57BL/6 mice, thus reversing the deleterious effects of ovariectomy on vertebral bone mass and microarchitecture [17]. Similarly, high-dose supplementation of resveratrol, a natural inducer of SIRT1, improves the BMD of male patients with obesity [18].

Sclerostin, the product encoded by the *SOST* gene, plays a key role in bone turnover by reducing osteoblasts’ differentiation and mineralization. As a result, *SOST* knockout mice exhibit an increased bone mass phenotype [19]. The *SOST* gene is an epigenetic target of SIRT1 in osteoblasts. *SOST* is negatively regulated through the deacetylation of its promoter at histone 3-lysine 9 induced by SIRT1 [20]. Interestingly, the treatment with the SIRT1 activator SRT3025 in mice reduces the expression of sclerostin in bone and increases the periosteal cortical mineralizing surface and the marker of bone formation pro-collagen type I. Analogously, SRT3025 downregulates sclerostin in a murine osteocyte-like Y4 cell line, whereas a reverse effect is observed with the SIRT1 inhibitor EX-527 [17].

In this scenario, the SIRT1/sclerostin pathway could represent an interesting way to evaluate the bone status in patients with different degrees of adiposity. Indeed, circulating SIRT1 shows a negative correlation with fat mass and a coherent trend with the well-recognized adipokines leptin and adiponectin [9,21], indicating a valid surrogate marker of adiposity; in contrast, sclerostin increases with fat [22,23,24]. Their mutual interaction still deserves to be clarified in relation to body composition and bone density and quality. Therefore, we aimed to assess the relationship between plasma SIRT1 and sclerostin and some parameters of bone status in different conditions of adiposity, comparing the bone in normal-weight, underweight and overweight patients.

## 2. Materials and Methods

### 2.1. Study Design and Population

Subjects enrolled were patients accessing the Centre of High Specialization for the Treatment of Obesity (CASCO) and the Day Service of the Department of Experimental Medicine, “Sapienza” University of Rome, Italy. Underweight subjects (patients with anorexia nervosa in medical stability), patients with obesity and normal-weight controls were recruited in the study.

Inclusion criteria were as follows: female gender, age ≤ 50 years old, pre-menopausal status and no acute disease that occurred in the three months preceding the enrollment. Exclusion criteria were as follows: pregnancy, secondary forms of osteopenia/osteoporosis due to endocrine diseases (primary hyperparathyroidism, hypogonadism, overt hyperthyroidism), rheumatological illness (rheumatoid arthritis), gastrointestinal malabsorption (celiac disease, lactose intolerance) and treatment with systemic or inhaled corticosteroids and furosemide. Patients with diabetes mellitus, chronic obstructive pulmonary disease, cancer, chronic renal failure and cirrhosis were also excluded. Physical examination, anthropometric measurements and history of previous low-trauma fractures were recorded for each patient. The study protocol was approved by the Ethical Committee of Sapienza University of Rome (C.E.) and conducted in accordance with the Declaration of Helsinki (1964) and subsequent amendments. All enrolled subjects signed a written informed consent form before entering the study; for minors, the consent was given by their parents.

### 2.2. Biochemistry and Kidney Function

Blood samples were collected between 8.00 and 10.00 a.m. after overnight fasting. Biochemical evaluation of bone metabolism included the following analytes: parathyroid hormone (PTH, n.v. 15–65 pg/mL), calcium (n.v. 8.4–10.8 mg/dL), phosphorus (n.v. 3.4–6.2 mg/dL), magnesium (n.v. 1.6–2.3 mg/dL), 25-OH-vitamin D (n.v. >30 ng/mL), alkaline phosphatase (ALP, n.v. 52–171 U/L), albumin (n.v. 35–55 g/L), urea (BUN, n.v. 10.2–49.8 mg/dL) and creatinine (n.v. 0.7–1.2 mg/dL). We, therefore, calculated corrected calcium for serum albumin concentration in order to obtain a more precise evaluation of the active proportion, and the estimated glomerular filtration rate (eGFR) with the Chronic Kidney Disease Epidemiology Collaboration (CKD-EPI) equation, which performs better than other equations at higher GFR, especially when actual GFR is >60 mL/min per 1.73 m^2^, with less bias and greater accuracy [25].

### 2.3. Plasma SIRT1 and Sclerostin Assay

Blood samples were collected in EDTA for plasma SIRT1 and sclerostin detection. After centrifugation, aliquots were collected and readily stored at −80 °C until analysis.

SIRT1 levels were determined using a commercial ELISA kit (GDMBS705558, MyBioSource), as previously described [10], with an inter- and intra-assay coefficient of variation of 10 and 8%, respectively, and a detection limit of 0.039 ng/mL.

The sclerostin concentration was measured through a commercial ELISA kit (Quantikine Human SOST Immunoassay ELISA kit (DSST00, R&D Systems, Minneapolis, MN, USA). The inter- and intra-assay coefficient of variation were 2% and 9.5%, respectively, the sensitivity was 1.74 pg/mL and no significant cross-reactivity or interference between this analyte and analogs was described.

### 2.4. Dual-Energy X-ray Absorptiometry and Trabecular Bone Score (TBS) Analysis

All subjects underwent BMD assessment via dual-energy X-ray absorptiometry (DXA) at the lumbar spine (L1–L4 anteroposterior) and hip (left femoral neck). All scans were administered by a trained technician using standardized procedures recommended by GE Healthcare. The instrument was calibrated daily using a phantom according to the manufacturer’s instructions (Hologic A Inc., Bedford, MA, USA, QDR 4500 W), as previously described [26]. BMD was recorded in terms of the absolute mineral content (in g/cm^2^) at various sites. Reduced mineral density diagnosis was made if the Z-score was <−2 SD (number of SD away from average value of age- and gender-specific reference group), as recommended by the International Society for Clinical Densitometry [27].

The integrated software TBS iNsight, version 2.1.2.0, was applied by the same technician to the site-matched spine scans for the evaluation of TBS, an indirect indicator of microarchitecture and bone quality estimation. We used the following cutoff points for TBS evaluation [28]: TBS > 1.350 as normal, TBS between 1.200 and 1.350 as indicative of partially degraded microarchitecture and TBS < 1.200 as degraded microarchitecture.

Whole-body fat mass (FM, %), abdominal visceral adipose tissue (VAT, cm^3^) and lean mass (kg) were also measured using the DXA technique. Body composition analysis of soft tissue was performed using the QDR2000 Product Software, version 13.5.3 A (Hologic, Middlesex County, MA, USA) and the regional limits were set as previously described [21].

### 2.5. Statistical Methods

Data are expressed as mean ± standard deviation (SD). Normality was assessed with the Shapiro–Wilk test, and data were log-transformed when appropriate. Differences between groups stratified by BMI (UW, NW, OB) were analyzed by performing ANOVA and post hoc analysis; the Mann–Whitney test was applied when amenorrhoeic and eumenorrhoeic subjects in the underweight group were compared. The Spearman correlation test was used to measure a linear univariate association between variables. BMD and TBS were analyzed as dependent variables among the independent parameters of age, BMI, SIRT1, sclerostin and trunk/legs FM ratio in a linear multivariate regression model. Multivariate stepwise regression analysis was used to identify predictors of BMD and TBS in the sample without underweight patients. Significance was accepted at an alpha level of <0.05 for all analyses. Statistical analysis was performed using SPSS Statistics for macOS, Version 25.0, Armonk, NY, USA: IBM Corp.

## 3. Results

### 3.1. Clinical Features and Differences between Groups Stratified by BMI

Sixty-six subjects who met the inclusion criteria were recruited for the study. Specifically, 16 underweight, 25 normal-weight and 25 obese subjects were enrolled. The demographic, anthropometric and clinical characteristics of the participants, stratified by BMI, are shown in Table 1. The mean age was 30.2 ± 10.7 years (range 15–50 years). Underweight patients were younger in comparison to the other groups (mean age 22.9 ± 9.4 years, *p* < 0.05). All patients were pre-menopausal, with regular menstrual cycles, except for eight underweight subjects, who had secondary amenorrhea. No low-trauma fractures were reported in the entire sample.

Among the biochemical bone metabolism parameters, ALP, BUN and magnesium did not differ between groups; creatinine, eGFR, albumin, calcium and phosphorus were within the normal range in all groups. As expected, the 25OHVitD levels decreased with the increase of BMI with a statistical significance between underweight and patients with obesity (*p* = 0.012). A difference in PTH was found between these two groups (*p* < 0.05); no patient with secondary hyperparathyroidism was present in the study. Glycemia was markedly higher in patients suffering from obesity relative to normal-weight (*p* = 0.001) and underweight subjects (*p* < 0.001).

Significant differences were observed among subgroups regarding total FM% (*p* < 0.001); trunk FM% (*p* < 0.001); abdominal VAT (*p* < 0.001); and trunk/legs FM ratio, which is an index of fat distribution (*p* < 0.001). In particular, the VAT area was significantly different between underweight and normal-weight patients (*p* < 0.05) and patients with obesity (*p* < 0.001). In line with the increase in FM%, SIRT1 concentrations showed a marked decrease in the group of patients with obesity when compared either to normal weight (*p* < 0.01) and underweight subjects (*p* < 0.001). In contrast, sclerostin increased significantly with weight and showed the highest values in the patients suffering from obesity when compared to the underweight and normal-weight subjects (*p* < 0.001).

We identified glycemia, SIRT1 and sclerostin as the biochemical parameters with the highest power regarding statistical differences (upper 95% with α set < 0.05) in a multivariate analysis in relation to the BMI categories. PTH, vitamin D, eGFR and calcium did not reach 80% power, while the other significant variables showed very mild power, not exceeding 65%.

Lumbar spine BMD, femoral neck BMD and lean mass also increased significantly with BMI. This may have been due to higher mechanical loads, as patients with obesity showed the highest levels of BMD (Table 1). Reduced bone mineralization was found in four underweight and two normal-weight subjects (Z-score < 2 standard deviations below age-matched individuals).

We observed lower values of TBS in both underweight patients and patients with obesity compared to normal-weight individuals, suggesting that compromised metabolic and nutritional conditions had a negative impact on trabecular bone microarchitecture (Table 1). There were no differences in spine BMD, femoral neck BMD or TBS in the underweight patients, irrespective of amenorrhea (data not shown).

### 3.2. Association between Body Composition and Circulating SIRT1 and Sclerostin

**Figure 1:**
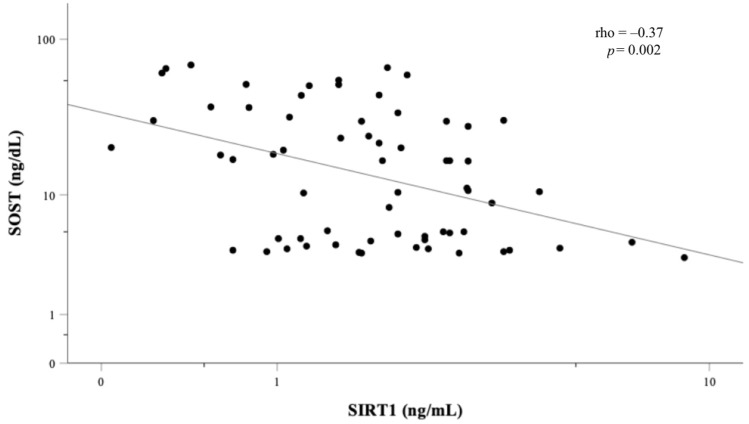
Bivariate correlation between circulating SIRT1 and sclerostin (SOST). Data were log-transformed. Correlation coefficient (ρ) and level of significance (*p*) are provided.

SIRT1 negatively correlated with BMI (ρ = −0.407, *p* = 0.001), weight (ρ = −0.391, *p* = 0.001), abdominal VAT area (ρ = −0.329, *p* = 0.009), total FM% (ρ = −0.485, *p* < 0.0001), trunk FM% (ρ = −0.451, *p* < 0.001) and serum glucose level (ρ = −0.302, *p* = 0.014) (Table 2). Conversely, there was a positive association between sclerostin and the adiposity indexes, in particular, with those of visceral fat apposition, such as abdominal VAT (ρ = 0.626, *p* < 0.0001) and trunk/legs FM ratio (ρ = 0.509, *p* < 0.0001). As expected, an inverse association between circulating SIRT1 and sclerostin was found (ρ = −0.37, *p* = 0.002) (Figure 1).

### 3.3. Correlation Analyses between Bone Parameters, Body Composition and Circulating SIRT1 and Sclerostin, and Multiple Regression Analyses for BMD and TBS Predictors

The BMD of the lumbar spine and femoral neck showed a significant, direct correlation with BMI (ρ = 0.602, *p* < 0.0001; ρ = 0.528, *p* < 0.0001, respectively), total FM% (ρ = 0.535, *p* < 0.0001; ρ = 0.475, *p* < 0.0001, respectively) and total lean mass (ρ = 0.579, *p* < 0.0001; ρ = 0.518, *p* < 0.0001, respectively). The impact of BMI on BMD and, concomitantly, on SIRT1 and sclerostin, revealed that SIRT1 was inversely correlated with the BMD of the lumbar spine and femoral neck (ρ = −0.381, *p* = 0.002; ρ = −0.275, *p* = 0.027, respectively), and that sclerostin was directly correlated with the BMD of the same bone districts (Table 2).

While SIRT1 and sclerostin did not reach a significant correlation with TBS (Table 2), TBS negatively correlated with weight (ρ = −0.314, *p* = 0.013), BMI (ρ = −0.329, *p* = 0.009), abdominal VAT (ρ = −0.392, *p* = 0.002), the trunk/legs FM ratio (ρ = −0.27, *p* = 0.03), glycemia (ρ = −0.37, *p* = 0.003), total FM% (ρ = −0.174, *p* = 0.17) and lean mass (ρ = −0.357, *p* = 0.005).

**Figure 2:**
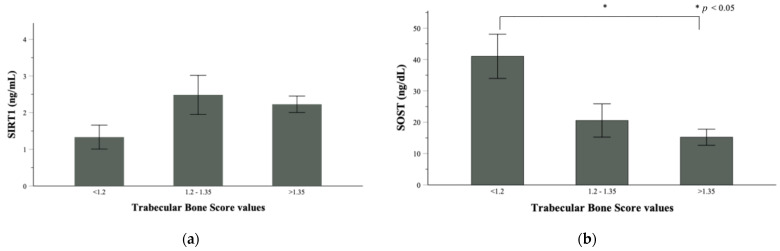
Distribution of (**a**) plasmatic SIRT1 and (**b**) sclerostin according to TBS categories. TBS < 1.2 indicates bone microarchitecture degradation, TBS between 1.2 and 1.35 indicates a partially degraded microarchitecture and TBS > 1.35 represents a normal microarchitecture.

After stratification for TBS, 10 patients showed a degraded microarchitecture (TBS < 1.200), 16 patients had a partially degraded microarchitecture (TBS between 1.200 and 1.350) and 40 patients had a normal trabecular bone microarchitecture (TBS > 1.350). Intriguingly, all 10 patients into the lowest range of trabecular bone quality suffered from obesity, 9 out of 25 patients with obesity had partially degraded microarchitecture and only 6 out of 25 patients with obesity had a normal TBS. Most of the normal-weight (22/25) and underweight patients (12/16) were in the normal TBS range (>1.350). It is noteworthy that underweight patients showed a better TBS than patients with obesity, suggesting a more detrimental effect of fat excess than fat deficiency on this parameter. The reduction of TBS was flanked by the reduction of SIRT1 (Figure 2a) and the significant increase of sclerostin (Figure 2b).

A multiple regression analysis was performed in order to identify the independent predictors of BMD and TBS (Table 3). Based on previous correlation results, along with variables considered important on clinical grounds, we included SIRT1, sclerostin age, BMI and the trunk/legs FM ratio in the analysis, with the latter being a representative index of fat distribution. Regression coefficients indicated BMI as the primary positive determinant for spine and femoral neck BMD and the negative determinant for TBS. SIRT1 was a negative predictor for spine BMD (Table 3).

### 3.4. Linear Correlation and Multiple Regression Analyses between BMI-Adjusted BMD and TBS and Circulating SIRT1 and Sclerostin

To correct for the influence of the weight load on bone, the spine BMD, femoral neck BMD and TBS were corrected for BMI by calculating their ratios, the mean values of which were 0.42 ± 0.12, 0.34 ± 0.10 and 0.58 ± 0.22, respectively. The subsequent correlation analysis showed a positive association of SIRT1 with the spine BMD/BMI ratio, femoral neck BMD/BMI ratio and TBS/BMI ratio (*p* < 0.05) and, accordingly, a significant negative correlation of sclerostin with the same BMI-adjusted skeletal sites (see Table 4).

The following regression analysis for the prediction of BMI-adjusted spine BMD, femoral neck BMD, and TBS highlighted the negative influence of sclerostin on spine and femoral neck districts and TBS (Table 5), and the positive predictive power of SIRT1 for TBS.

### 3.5. Linear Correlation and Regression Analyses between SIRT1, Sclerostin and Bone Parameters among Normal-Weight and Obese Patients

We performed complementary analyses excluding the underweight group in order to evaluate the relationship between adiposity and bone without the interferences due to underweight malnutrition, low patient age and hypoestrogenism on mineral density.

Bivariate correlations confirmed the inverse association of SIRT1 with sclerostin (ρ = −0.395, *p* = 0.005). When structural bone parameters were standardized for BMI, SIRT1 revealed significant positive correlation with L1–L4 BMD (ρ = 0.358, *p* = 0.011), femoral neck BMD (ρ = 0.383, *p* = 0.007) and TBS (ρ = 0.388, *p* = 0.008); conversely, sclerostin confirmed its negative correlation with lumbar BMD (ρ = −0.328, *p* = 0.023), femoral neck BMD (ρ = −0.399, *p* = 0.005) and TBS (ρ = −0.466, *p* = 0.001).

Multivariate stepwise regression analysis was used to identify factors that influence BMD and TBS across the obese and normal-weight groups. The regression included age, SIRT1 and sclerostin and, although the results were limited by the small sample size, the results provided the set of independent variables that best explained the variance in bone in the current sample. The analysis confirmed that SIRT1 was a positive determinant of TBS (ρ = 0.310, *p* = 0.016) that, together with age (ρ = −0.480, *p* < 0.001), explained 41% of the TBS. Sclerostin remained the best negative determinant for BMD at the femoral site (β = −0.449, *p* = 0.001).

## 4. Discussion

Obesity and osteoporosis are two of the most common chronic diseases of our time, representing a significant public health challenge [1,29]. Over the last few decades, many research efforts have been dedicated to understanding whether changes in weight and fat mass could contribute to the pathophysiology of bone. Cells of the adipocyte lineage, as well as cells of the osteoblast lineage, are increasingly identified as participants in whole-body homeostasis and metabolism. Therefore, the relationship between adipose tissue and bone, whose cells arise from the same progenitor, provides insight into the pathophysiology of both adiposopathy and bone mass loss and degradation [30,31].

Adipocytes secrete cytokines and adipokines that either stimulate or inhibit osteoblasts [32]. Moreover, in obesity, excess adipose tissue becomes dysfunctional, promoting a pro-inflammatory, hyperlipidemic and insulin-resistant environment through the release of several inflammatory cytokines and chemokines [33].

It has been recognized that tissue SIRT1 and sclerostin influence bone health and are involved in the bone remodeling process and maintenance of bone biomechanical characteristics. Their reciprocal interaction in relation to the degree of adiposity and their functional correlation still need to be clarified. Through epigenetic regulation, SIRT1 modulates senescence processes, cell circadian rhythm and apoptosis; reflects the beneficial effects of calorie restriction; and protects against aging-related diseases, including bone mass loss [8]. As a nutrient-sensitive regulator of bone remodeling, SIRT1 is associated with bone mass gain [16], and activation of SIRT1 decreases adipocyte formation during the osteoblast differentiation of mesenchymal stem cells [15]. SIRT1-null mice show a low bone mass phenotype [8,13,34] and delays in mineralization of the skull, vertebrae and digits associated with craniofacial abnormalities [35]. Conversely, SIRT1 transgenic mice are protected against age-induced osteoporosis [8], and mice treated with SIRT1 agonists show enhanced BMD [36].

To our knowledge, this is the first study describing the relationship between blood SIRT1 and sclerostin with bone health indexes among patients with different adiposity levels. We found that circulating SIRT1 and sclerostin were linked by an inverse association across different fat mass degrees and that they had a consistent relationship with the bone mass. Since the expression of both SIRT1 and sclerostin is strongly influenced by the fat mass and, in turn, the mechanical load is crucial for BMD, a complex relationship between these molecules and bone depending on adiposity is predictable.

**Figure 3:**
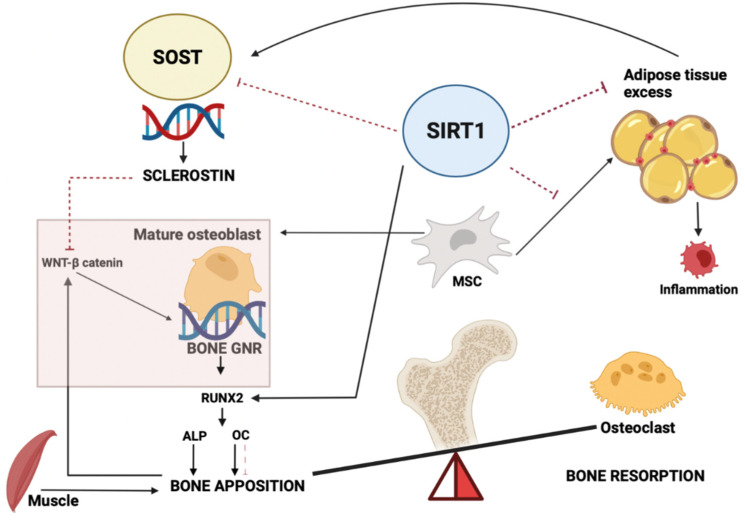
Effects of SIRT1 and sclerostin on bone. SIRT1 can determine the fate of mesenchymal stem cells by promoting osteogenic differentiation and suppressing adipogenic differentiation. SOST/sclerostin expression is controlled by epigenetic modifications and transcriptional regulation induced by SIRT1. Abbreviations: SIRT1, sirtuin 1; GNR, genetic regulatory network; RUNX2, runt-related transcription factor 2; ALP, alkaline phosphatase; OC, osteocalcin; MSC, mesenchymal stem cells.

Recently, Chekroun and colleagues provided a review of empirical evidence on the complex bone genetic regulatory network (GRN) that directly influences bone protein production and bone mineralization [37]. In such a complex scenario, SIRT1 plays an important role in regulating the processes of osteoblastogenesis by integrating multiple upstream and downstream signals. As shown in Figure 3, SIRT1 is a positive regulator of the main osteoblast transcription factor RUNX2 [38]. In addition, among others [15,16,38,39], the *SOST*/sclerostin system is a crucial one [20]. Sclerostin is secreted by the osteocytes, which recruit osteoblast precursors from mesenchymal cells. Mature osteoblasts then regulate bone remodeling processes under the influence of hormones and several growth factor signals [40,41]. Sclerostin inhibits osteoblast differentiation and, consequently, bone mineralization by interacting with the Wnt/β-catenin pathway, which represents one of the fundamental mechanisms of signaling that promotes cell proliferation, cell polarity and osteogenesis in humans [42]. Loss-of-function mutations in the *SOST* gene are associated with high bone mass [43], and the sclerostin antibody *Romosozumab* is a recently approved drug for the therapy of postmenopausal osteoporosis [44]. Additionally, a study by Shu Ma et al. showed that microRNA-96, which is capable of binding and inactivating sclerostin, led to the Wnt signaling pathway activation with subsequent osteoblast differentiation in mice; vice versa, inhibition of miR-96 resulted in the suppression of bone formation [45]. Sclerostin is negatively regulated by SIRT1, which tightly controls its expression via epigenetic modifications on its promoter (Figure 3). In accordance with tissue studies data [17,20], we confirmed this inverse correlation between circulating SIRT1 and sclerostin, which could therefore be traced to the dual mechanism of increased sclerostin production by osteocytes and the lack of inhibitory effect of SIRT1 on *SOST* in the case of weight excess.

Like SIRT1, sclerostin also intervenes in metabolic processes and the regulation of fat mass amount and visceral fat apposition. While SIRT1 decreases with increasing body fat [9], sclerostin expression increases in parallel with adipose tissue [23] and insulin resistance [46], and promotes adipocyte hypertrophy [19,47] and the differentiation of bone precursors into white adipocytes, reducing bone formation. In aging male mice, oxidative conditions aggravate bone loss by downregulating SIRT1 and increasing SOST mRNA and protein [48]. Accordingly, either *SOST*-knockout mice or sclerostin-neutralizing antibody-treated mice exhibit a phenotype characterized by increased bone mass and reduced white adipose tissue [19]. Ross et al. reported decreased bone formation, decreased osteocalcin expression and increased sclerostin RNA expression in young and old HFD-fed mice compared to standard chow-fed mice, confirming the negative effect exerted by metabolic diseases on bone, including through the activation of sclerostin [49].

In line with these results, we found that the levels of sclerostin increased in parallel with FM%, abdominal VAT, trunk/legs FM and glycemia, and that plasma SIRT1 behaved in the opposite way.

Several studies showed that preserved cortical bone status and strength are associated with a lower risk of major adverse coronary events [50]. Interestingly, increased levels of sclerostin were found both in serum and bone specimens of patients with type 2 diabetes [51,52], post-menopausal women compared to pre-menopausal ones [22,53], immobilized postmenopausal women with bone resorption markers elevation [54] and postmenopausal women with osteoporosis compared to postmenopausal women without osteoporosis, even if no correlation was seen with BMD [55]. These findings could suggest that elevated sclerostin levels are often seen in subjects with increased cardiovascular risk and with bone loss. Indeed, we found the highest blood sclerostin concentrations in patients with obesity, who are metabolically more compromised compared to underweight and normal-weight individuals, suggesting that the concomitant increase of plasma sclerostin with adiposity might be involved in the worsening of bone quality in subjects with obesity. Intriguingly, beyond the difference in adipose tissue, BMI categories were characterized more by the different concentrations of blood SIRT1, sclerostin and glucose than by the different concentrations of other biochemical parameters. We found higher BMD values in the group of patients affected by obesity. Mechanical stress is an important factor affecting bone remodeling and strength [4], and BMD transiently increases with weight gain and eventually reduces with weight loss [56]. This characteristic of bone homeostasis has often run into misleading evidence of the paradoxically beneficial effect of weight on bone. The phenomenon known as the obesity paradox is confirmed by our results. BMI was the strongest predictor of BMD in our cohort, despite the lower levels of SIRT1 and the higher levels of sclerostin seen in the group of patients with obesity should have predicted a negative effect on bone. Some studies on sclerostin and bone reported this condition [23,57,58,59] and, in some cases, higher serum sclerostin was measured in women with normal BMD compared to women with osteoporosis [23]. However, the subtle relationship between excess fat, weight load and skeletal mass could be made more complex by the impaired adipokine production being secondary to increased fat mass [21]. Indeed, an increased risk of fragility fractures, despite a normal BMD, has been associated with secondary factors [60]. On the other hand, the high sclerostin levels with low SIRT1 in our cohort of patients with obesity fit with the significant downregulation of osteoblasts’ SIRT1 following mechanical stress [61].

Therefore, a reasonable hypothesis when evaluating the relationship between all these elements that contribute to establishing the strength of the bone is to correct the BMD with respect to adiposity. In light of the aforementioned considerations, we calculated the ratio of BMD to BMI to better evaluate the relationship between SIRT1, sclerostin and bone mass. After such an adjustment, we consistently observed a congruent correlation of SIRT1 and sclerostin with the BMD of the spine and femoral neck. Taken together, these results suggested that the ratio of BMD to BMI could be an easily achievable index that allows for bone analysis through the reduced interference of body weight. This could be particularly useful when evaluating patients with obesity, who fracture much more often than would be expected from their BMD values.

To get a more truthful picture of bone quality, we extended the bone assessment to TBS. A trabecular bone is a large-surface bone widely exposed to blood flow and bone marrow that is differentially influenced by several biochemical factors, including hormones and drugs, and with a higher turnover compared to dense cortical bone [7]. For these reasons, the metabolic imbalance can easily lead to its microarchitectural deterioration and worsening of mechanical properties [62,63]. TBS, determined from lumbar spine DXA images, reflects qualitative aspects of the skeletal structure, providing an indirect index of trabecular microarchitecture that is partially independent of, but complementary to, BMD [64]. Although its use in the extreme ends of BMI is questioned due to the possible source of bias of too little or too much adipose tissue interposed between the spine and the X-ray, TBS has become a reference tool for skeletal assessment, and several studies support its reliability and reproducibility in clinical practice, both in underweight [65] and obese populations [66,67]. We found that most of the patients with obesity had the lowest value of TBS, confirming that the worse the metabolic state, the greater the suffering of the trabecular bone [68,69]. Indeed, TBS was negatively correlated with biological parameters of adiposity, such as abdominal VAT, the trunk/legs FM ratio and blood glucose. Moreover, the lower the TBS/BMI ratio, the lower the SIRT1 levels and the higher the sclerostin levels, consistent with the finding that increased sclerostin expression is detrimental for bone [70]. Accordingly, after stratification for TBS, we observed that all the patients with the lowest TBS were obese and had higher sclerostin and lower SIRT1.

In general, there are no conclusive data as to where secreted SIRT1 is coming from (i.e., a particular tissue or cell type) during fasting or CR. However, recent findings report that circulating levels of SIRT1 measured in plasma are linked to visceral adipose tissue in mice [71]. Moreover, some authors measured SIRT1 activity in peripheral blood, giving evidence that the measurement of blood SIRT1 levels may reflect SIRT1 tissue levels [11,72,73].

As already described, we found the highest levels of SIRT1 in the underweight group of patients [10,21]. Accordingly, mice under calorie restriction with a significant loss of body weight show the upregulation of SIRT1 mRNA expression in bone associated with increased bone mass [16]. Conversely, despite the higher SIRT1 and the lowest sclerostin levels, possibly due to the inhibitory effect of SIRT1 on sclerostin, the underweight patients showed the lowest BMD. We are aware that a large number of variables may have influenced this data, including the reduced mechanical load, the undernourishment [74] and the amenorrhea of some of the study participants, although no differences in BMD were observed between amenorrhea and normal menstruating patients [40,75]. In addition, the young age of the underweight patients also partly explains this finding, as peak bone mass is typically reached in early adulthood [76].

Nevertheless, when the underweight group was excluded from our analysis, SIRT1 was confirmed to be a positive predictor for TBS, while sclerostin maintained its negative prediction for femoral neck BMD. This suggests that, after removing some confounding conditions, the SIRT1/sclerostin interplay remained crucial for bone homeostasis. Although BMD is still considered a major determinant of bone strength, fragility fractures are reported only from a limited percentage of patients affected by anorexia and low BMD [77]. This evidence, together with the fact that most individuals with a fragility fracture show BMD value in the osteopenic or even in the normal range [60], indicates that other factors participate in determining bone strength and fracture risk beyond BMD and TBS. The inhibition of sclerostin by SIRT1 could be one of those factors that allow subjects with low BMD and nutritional deficiencies, or even with a lack of estrogens, to be protected against fragility fractures. Moreover, the significantly better TBS of underweight patients compared to patients with obesity could be interpreted as more evidence in support of the beneficial effect of high SIRT1 and low sclerostin pattern on bone microarchitecture and quality.

We are aware that our study has several limitations. Malnutrition/malabsorption, vitamin deficiencies, hormonal alterations, reduced bone load, young age and peak bone mass not yet reached make it difficult to compare the bone mass of underweight patients with those of normal-weight or obese patients. The inclusion of underweight patients is problematic; thus, we performed complementary analyses excluding this population. Nevertheless, underweight patients still represent a valuable model to compare both the different degree of adiposity and its sequelae and how fat mass amount impacts the expression of SIRT1 and sclerostin.

The sample size of our study was relatively small, possibly hindering some of the results, and the lack of male patients did not allow for the results to be extended to both sexes. Although we deliberately recruited female patients under 50 years of age to avoid advanced age and estrogen deprivation due to menopause as an additional source of bias, this did not allow us to evaluate the behavior of SIRT1 and sclerostin in an osteoporotic population. Finally, the lack of some covariates of BMD and TBS in the adjusted analyses (e.g., physical activity, smoking, alcohol), including further bone turnover markers, are considered additional shortcomings or weaknesses that warrant further, adequately powered, confirmatory studies.

The primary aim of the present study was to evaluate and compare the circulating SIRT1 and sclerostin relationship with bone mass and quality, taking into account their dependence on the degree of adiposity. Our results show that, even after the exclusion of underweight patients from the sample, SIRT1 maintained a positive role in regulating TBS, the bone structure most exposed to the effects of metabolic disorder, and sclerostin has a negative impact on BMD. SIRT1 and sclerostin are ideal candidates for evaluating the crosstalk between adipose tissue and bone, and their circulating form allows us to study these relationships without carrying out invasive sampling.

## 5. Conclusions

There is growing evidence that some factors released by the adipose tissue into the bloodstream may have detrimental effects on bone mass and, conversely, the reduced release of certain factors decreases beneficial effects on the bone. The paracrine and endocrine functions of adipose tissue and bone are essential for overall homeostasis, beyond their classic roles. We highlighted areas in which circulating SIRT1 and sclerostin yield additional insights into the pathophysiology of bone and adipose tissue interactions. Although the significance of blood SIRT1 and sclerostin remains to be fully elucidated, our findings expand previous data supporting the existence of bone–adipose tissue crosstalk and the potential of new plasma biomarkers for bone risk stratification. SIRT1 and sclerostin are emerging key players of the bone–adipose tissue crosstalk, as well as intriguing pharmacological targets for the treatment of fat mass excess and bone mass loss.

## Figures and Tables

**Table 1:** Demographic, anthropometric and clinical characteristics of the study patient population.

	Underweight	Normal Weight	Obesity	*p*-Value
*n*	16	25	25	-
Age (years)	22.9 ± 9.4	28.5 ± 6.5	36.5 ± 11.6	<0.001
Weight (kg)	44.3 ± 10.1	65.8 ± 8.5	104.1 ± 15.6	<0.001
BMI (kg/m^2^)	16.7 ± 2.8	22.5 ± 1.7	38.7 ± 4.2	<0.001
**Biochemistry**				
SIRT1 (ng/mL)	3.07 ± 2.3	2.5 ± 0.9	1.27 ± 0.87	<0.001
Sclerostin (ng/dL)	5.05 ± 1.5	15.3 ± 11.4	35.9 ± 20.6	<0.001
Glucose (mg/dL)	73.2 ± 6.6	81.8 ± 7.8	89.9 ± 7.8	<0.001
Creatinine (mg/dL)	0.69 ± 0.11	0.79 ± 0.12	0.73 ± 0.13	<0.05
BUN (mg/dL)	27.2 ± 7.7	33.1 ± 9.77	29.5 ± 8.0	ns
eGFR (mL/min/1.73 m^2^)	123.5 ± 9.7	114.2 ± 9.2	110.5 ± 15.0	<0.01
ALP (U/L)	61.6 ± 17.7	53.8 ± 12.6	64.3 ± 18.1	ns
Calcium (mg/dL)	9.8 ± 0.4	9.5 ± 0.4	9.4 ± 0.4	<0.01
Phosphorus (mg/dL)	3.9 ± 0.5	4.0 ± 0.6	3.5 ± 0.6	<0.05
Magnesium (mg/dL)	2.1 ± 0.2	2.0 ± 0.1	2.0 ± 0.1	ns
25OHVitD (ng/mL)	37.3 ± 14.6	29.6 ± 11.2	24.7 ± 13.7	<0.05
PTH (pg/mL)	30.4 ± 8.3	35.6 ± 15.7	48.5 ± 23.9	<0.01
**Body Composition**				
Total FM (%)	19.7 ± 7.0	26.6 ± 6.5	41.0 ± 4.2	<0.001
Trunk FM (%)	14.1 ± 6.7	22.2 ± 7.0	39.1 ± 4.3	<0.001
Abdominal VAT (cm^2^)	15.2 ± 10.1	46.7 ± 21.4	120.8 ± 48.4	<0.001
Trunk/legs FM ratio	0.69 ± 0.2	0.9 ± 0.3	1.24 ± 0.3	<0.001
Lean mass (kg)	33.4 ± 5.3	46.0 ± 6.9	59.2 ± 10.4	<0.001
**DXA Bone Parameters**				
L1–L4 BMD (g/cm^2^)	0.87 ± 0.10	1.04 ± 0.14	1.14 ± 0.14	<0.001
Femoral neck BMD (g/cm^2^)	0.72 ± 0.11	0.85 ± 0.11	0.9 ± 0.15	<0.001
TBS	1.38 ± 0.06	1.42 ± 0.06	1.29 ± 0.14	<0.001

Abbreviations: BMI, body mass index; SIRT1, sirtuin 1; PTH, parathyroid hormone; Mg, magnesium; 25OHVitD, 25-hydroxyvitamin D3; ALP, alkaline phosphatase; eGFR, estimated glomerular filtration rate; BUN, blood urea nitrogen; FM, fat mass; VAT, visceral adipose tissue; BMD, bone mineral density; TBS, trabecular bone score. Values are expressed as mean ± standard deviation (SD). ns: no significance.

**Table 2:** Linear correlation analyses between SIRT1 and sclerostin and indicators under consideration.

	SIRT1		Sclerostin	
	ρ	*p*-Value	ρ	*p*-Value
Age (years)	−0.126	0.31	0.494	<0.001
BMI (kg/m^2^)	−0.407	0.001	0.596	<0.001
Glycemia (mg/dL)	−0.302	0.014	0.481	<0.001
Total FM (%)	−0.485	<0.001	0.504	<0.001
Abdominal VAT (cm^2^)	−0.329	0.009	0.626	<0.001
Trunk/legs FM ratio	−0.206	0.098	0.509	<0.001
Lean mass (kg)	−0.210	0.096	0.499	<0.001
Spine BMD (g/cm^2^)	−0.381	0.002	0.480	<0.001
Femoral neck BMD (g/cm^2^)	−0.275	0.027	0.292	0.019
TBS	0.032	0.806	−0.142	0.274

Abbreviations: SIRT1, sirtuin 1; BMI, body mass index; FM, fat mass; VAT, visceral adipose tissue; BMD, bone mineral density; TBS, trabecular bone score. Spearman correlation coefficients and *p*-values are indicated.

**Table 3:** Multiple regression analysis for best predictors of lumbar spine BMD, femoral neck BMD and TBS.

	Lumbar Spine BMD		Femoral Neck BMD		TBS	
	β	*p*-Value	β	*p*-Value	β	*p*-Value
Age (years)	0.107	0.42	−0.136	0.34	−0.197	0.18
SIRT1 (ng/mL)	−0.236	0.049	−0.173	0.17	−0.228	0.08
Sclerostin (ng/dL)	0.082	0.56	−0.073	0.64	−0.006	0.97
Trunk/legs FM ratio	−0.033	0.82	−0.092	0.56	−0.020	0.90
BMI (kg/m^2^)	0.379	0.019	0.578	0.001	−0.462	0.014

Abbreviations: BMD, bone mineral density; TBS, trabecular bone score; SIRT1, sirtuin 1; FM, fat mass; BMI, body mass index. β coefficients and *p*-values are indicated.

**Table 4:** Correlation analysis between SIRT1 and sclerostin and BMI-adjusted BMD and TBS.

	SIRT1		Sclerostin	
	ρ	*p*-Value	ρ	*p*-Value
Spine BMD/BMI ratio	0.320	0.009	−0.472	<0.001
Femoral neck BMD/BMI ratio	0.326	0.008	−0.526	<0.001
TBS/BMI ratio	0.394	0.002	−0.598	<0.001

Abbreviations: SIRT1, sirtuin 1; BMI, body mass index; BMD, bone mineral density; TBS, trabecular bone score. Spearman correlation coefficients and *p*-values are indicated.

**Table 5:** Multiple regression analysis for the prediction of BMI-adjusted BMD and TBS.

	Spine BMD/BMI		Femoral Neck BMD/BMI		TBS/BMI	
	Β	*p*-Value	β	*p*-Value	β	*p*-Value
Age (years)	−0.181	0.175	−0.333	0.008	−0.280	0.015
SIRT1 (ng/mL)	0.119	0.316	0.096	0.375	0.219	0.033
Sclerostin (ng/dL)	−0.353	0.014	−0.347	0.008	−0.402	0.001

Abbreviations: BMD, bone mineral density; TBS, trabecular bone score; SIRT1, sirtuin 1; BMI, body mass index. β coefficients and *p*-values are indicated.

## Data Availability

The datasets generated for this study are available on request to the corresponding author. Requests to access the datasets should be directed to s.mariani@uniroma1.it.
